# Understanding the contribution of primary and community services to health system resilience during the COVID19 Pandemic in Aotearoa, New Zealand: a qualitative interview study

**DOI:** 10.1186/s12913-024-12078-6

**Published:** 2024-12-24

**Authors:** Vanessa Burholt, Janine Wiles, Alison Schneller

**Affiliations:** 1 School of Nursing/School of Population Health, Faculty of Medical and Health Sciences, University of Auckland, Room 235B, Building 505, 85 Park Road, Private Bag 92019, Grafton, Auckland, New Zealand; 2 Faculty of Medicine, Health and Life Science, Swansea University, Singleton, Swansea, Wales; 3 Social and Community Health, School of Population Health, Faculty of Medical and Health Sciences, University of Auckland, Auckland, New Zealand; 4 School of Nursing, Health, Faculty of Medical and Health Sciences, University of Auckland, Auckland, New Zealand

**Keywords:** Emergency preparedness, Health system resilience, Community services, Older people, COVID-19, Aotearoa New Zealand

## Abstract

**Background:**

The COVID-19 pandemic exposed critical gaps in health system preparedness. This study, guided by a critical ecological model, examines the experiences of primary health and community services in Aotearoa New Zealand during the pandemic, focusing on their response to older people and their unpaid caregivers. The study aims to identify effective strategies for health system resilience. It addresses the question, what can we learn from the experiences of organisations supporting older people and caregivers during COVID-19, to prepare for other similar (emergency health) situations?

**Methods:**

A multidisciplinary research team conducted cross-sectional qualitative research through semi-structured telephone interviews with service providers (SPs) delivering primary or community home-based services to older people and caregivers across Aotearoa New Zealand. SPs included national organisations, Māori, Pacific, or rural providers and dementia services. Data were collected between July and October 2020. Notes were taken during interviews using a Rapid Appraisal Procedure grid, which were later revised and validated by participants. Data were analysed using a hybrid deductive-inductive thematic analysis, following COREQ guidelines.

**Results:**

Twenty staff (Chief Executive Officers and representatives) from national organisations (*N* = 4), Māori (*N* = 3), Pacific (*N* = 5), rural (*N* = 4), and regional dementia (*N* = 4) SPs were interviewed. SPs demonstrated resilience through collaboration, adapting delivery models, and upskilling staff. Key challenges involved inconsistent identification of vulnerable clients, limited access to aged residential care, and barriers relating to digital access which disproportionately affected older adults and staff, and clients in rural areas. Workforce shortages, and unclear public policy concerning travel across regions further complicated service delivery, highlighting the interaction between policy, community, interpersonal, and individual factors.

**Conclusions:**

Aotearoa New Zealand managed COVID-19 effectively, but the pandemic exposed areas for improvement in health system resilience. The government demonstrated absorptive resilience through swift actions, including lockdowns and clear communication, while SPs exhibited adaptive resilience by modifying service protocols. Knowledge gained from this study can contribute to transformative resilience. Long-term strategic changes are necessary to improve emergency planning, such as developing a unified framework to inform a ‘Priority List’, enhancing workforce capacity, and addressing digital exclusion. These steps can strengthen health system robustness and preparedness for future crises.

**Supplementary Information:**

The online version contains supplementary material available at 10.1186/s12913-024-12078-6.

## Background

COVID-19 was categorised as an unexampled event, because it differed significantly from previous pandemics in several ways and was beyond the individual or collective experience of health systems [1]. The speed and breadth of transmission were unprecedented, amplified by air travel, urbanization, and interconnected economies. While earlier pandemics had overwhelmed healthcare systems, COVID-19 did so on a global scale, pushing advanced healthcare infrastructures to their limits and leading to severe shortages of medical supplies, hospital beds, and ventilators, creating ethical dilemmas around patient care [2, 3]. Lockdowns and travel restrictions caused global economic disruption, with industries halted, increased living costs, and widespread unemployment [4]. Furthermore, the role of social media during the pandemic played a major part in shaping public perception [5]. While health systems are expected to have emergency preparedness protocols in place, a review of COVID-19 preparedness and response planning documents from 106 countries showed that less than half had plans that considered maintaining essential health services (47%), and only 34% considered subnational service delivery [6].

In general, it is unreasonable to expect organisations to be prepared for unexampled events [7]. However, some health systems exhibited more resilience than others during the COVID-19 pandemic, demonstrating that there are different levels of preparedness for the unexpected. Some health systems suffered substantial (human) losses through COVID-19 [8], others witnessed organisations slowly or suddenly ‘fail’ [9]. Learning about the capacity of some primary health and community services to rebound after unanticipated events, can inform disaster preparedness and health system resilience [10]. This article draws on the experiences of primary health and community services responding to the needs of unpaid caregivers and older care recipients during the first waves of the COVID-19 pandemic in Aotearoa New Zealand to share relevant strategies and issues and contribute to future readiness to deal with similar events.

### Health system resilience

System resilience is operationalised as sustaining “operations under expected and unexpected conditions, by adjusting functioning prior to, during, or following changes, disturbances and opportunities”[7]. In health care systems, resilience is “the capacity to adapt to challenges and changes at different system levels, to maintain high quality care” [11]. A resilient health system requires a well-integrated range of organisations which can monitor the situation, learn and change practices, strategies, or protocols (e.g. during an emergency) and use this learning to improve and anticipate future events [11]. These organisations should have a good understanding of the diverse needs of clientele, and the various assets and resources of the health system including service delivery, a paid health workforce, and the ‘invisible’ workforce of unpaid caregivers.

Health system resilience can be understood as absorptive, adaptive, and transformative [12]. Absorptive resilience is the ability of a health system to withstand and manage the immediate impacts of a shock without major changes to its structure or functioning, maintaining core functions and services, utilising existing resources, and implementing emergency measures to buffer impact. Adaptive resilience refers to a health system’s capacity to adjust and reorganise in response to changing conditions and new information during a crisis, including learning and evolving practices to improve response and recovery efforts, optimising resources, and making flexible decisions to address emerging challenges. Transformative resilience is a health system’s ability to fundamentally change its structure and operations to better cope with future shocks and stresses, including long-term strategic changes addressing underlying vulnerabilities, enhancing system capacities, integrating innovations, and improving overall system robustness and sustainability.

Each type of resilience plays a crucial role in ensuring health systems survive and thrive in the face of crises. Exploring the different types of resilience, practices and experiences of discrete segments of national health care systems, such as service delivery and health workforces in primary health and community services during the COVID-19 pandemic, can inform future disaster preparedness and health system resilience [13].

Between April-September 2020, Te Hiringa Hauora / the Health Promotion Agency in Aotearoa New Zealand established a working group to develop approaches to address needs of older people as part of the Ministry of Health’s COVID-19 response. People aged 70 and older were issued with specific instructions to restrict social interactions (Table 1). For Māori and Pasifika communities, the age threshold for being considered ‘older’ was ≥ 60 years recognising of the earlier onset of age-related health conditions and lower life expectancy in these populations. The working group highlighted gaps in support for unpaid caregivers to older people, particularly for those living in rural areas, supporting people living with dementia, and of Māori and Pacific ethnicity. This led to the development of a research study *Health Equity And Wellbeing Among Older People’s Caregivers During COVID-19* [14].

**Table 1:** Description of restrictions for the general population, older adults, primary care, community care and residential and nursing aged care facilities during COVID-19 Alert Levels in Aotearoa New Zealand 2020

	**General Population**	**Older people**	**Primary Care**	**Health & Disability Community Services**	**Residential care facilities**
1	Contact tracing of cases. General hygiene practices. Public health campaigns to raise awareness	Normal activities with caution: advised to remain vigilant about hygiene and physical distancing	No restrictions, use of telehealth where appropriate, safety and hygiene practices	No restrictions, use of telehealth where appropriate, safety and hygiene practices	Normal visiting, health screening and infection control practices
2	Physical distancing in public and workplaces. Limits on gatherings. Encouraged to work from home where possible. Heightened tracing and testing. Closure of schools and non-essential businesses considered	Caution going out and attending small gatherings. Encouraged outdoor activities and exercise avoiding crowded places and maintaining physical distancing	Mixed model of in-person and telehealth consultations, with in-person visits following public health guidelines	Most services delivered with safety measures, including social distancing and hygiene practices	Controlled visitor access with restrictions, such as limited visitor numbers and health screening
3	Strict physical distancing and restrictions on public gatherings. Closure of public venues. Schools and childcare centres open only for children of essential workers. Essential services open, non-essential businesses and services closed. Travel restrictions within regions	Stay home and limit outings. Use support networks for essential supplies and medications. Short walks and outdoor exercise permitted, with physical distancing. Limited visits: visitors to adhere to strict hygiene and physical distancing protocols	Primarily via telehealth, with in-person visits allowed for urgent cases following strict safety measures	Essential services, some non-essential services with strict safety measures	Restricted visitor access, with some allowances for close family members under controlled conditions
4	Stay at home, except for essential personal movement. Severe restrictions on travel and movement. All gatherings cancelled, and all public venues closed. Schools and educational facilities closed. Only essential businesses and services open	Avoid all non-essential outings. Indoor exercises, short walks (only if necessary) maintaining physical distancing. Utilise support networks for essential supplies. No visitors allowed	Prioritised pandemic response Operated for urgent and essential services only. Routine consultations moved to telehealth wherever possible	Essential services with significant restrictions. Non-essential services paused	Strictly no visitors, except for end-of-life care. Stringent infection control measures

The study was developed to examine the intersectionality of structural inequities, focusing on subgroups of caregivers who may face particular challenges [15]. One of the study objectives was to undertake interviews with organisations providing services to caregivers and older people (service providers (SPs)) to examine health system resilience in this sector. This article focuses on this specific element and seeks to address following question:

*What can we learn from the experiences of organisations supporting older people and caregivers during COVID-19, to prepare for other similar (emergency health) situations?*

**Fig. 1:**
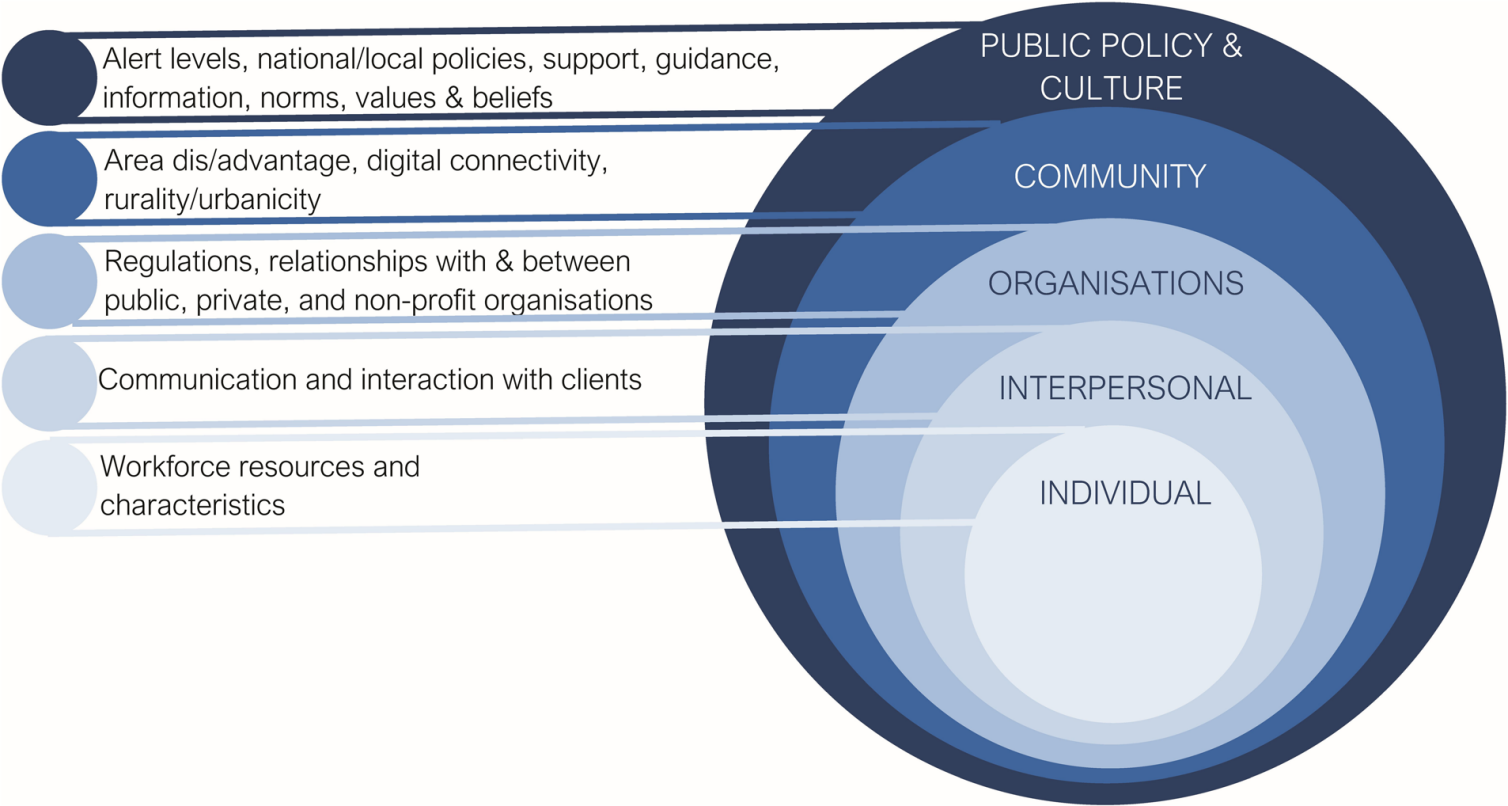
Critical ecological model comprising inter-related systems that influence health system resilience

A critical human ecological model provided a meta-framework for study [16]. The five integrated levels of influence in the critical human ecological model (Fig. 1) comprise public policy and culture (laws and policies that regulate and support health systems, alongside beliefs, values, norms, customs, practices in society), communities (the broad social settings in which service delivery occurs), organisations (relationships with and between public, private, and non-profit service provider organisations, and the regulations and protocols that organise behaviours), interpersonal (communications and engagement between organisations and their clients), and individual (resources and characteristics of the organisational workforce) elements.

A critical human ecological perspective situates the dynamics of organisational capacities, disparities in access to health resources, or workforce fatigue within broader policy contexts, examining how governance at local, national, and global levels either bolstered or undermined health system resilience during the pandemic [17].

## Methods

### Study design

The study used a cross-sectional qualitative study design to address the research question. It follows the COnsolidated criteria for REporting Qualitative research (COREQ) Checklist for rigour [18].

### The study setting

The study was undertaken in Aotearoa New Zealand. The country has an estimated population of 5 million people comprising 67.8% European, 19.6% Māori (indigenous population), 17.3% Asian and 8.9% Pacific peoples [19]. The health system in Aotearoa New Zealand is publicly funded (through taxation) and primarily government-operated; users typically pay a surcharge for access to primary care. A complementary private health sector offers additional services and often faster access to elective surgeries and specialist care (see Additional File 1). However, the country has an ageing population, and the health system is reliant on the unpaid contributions of caregivers. A majority of physical, emotional and practical support for sick, disabled or older people is provided by unpaid caregivers [20], with approximately 480,000 people providing regular care for someone with an illness or disability [21]. The health care system has a strategic role to play in promoting health equity [22].

### Interviews and recruitment

Participants were CEOs or the nominated representative of an eligible service provider organisation, aged over 18 years, and able to communicate verbally in English. Eligible organisations were those that provided services to unpaid caregivers or older people in the community during alert levels 2–4. They were either national organisations, Māori providers, Pacific providers, rural providers (i.e. serving a population dwelling in rural areas), or local or regional providers focusing on dementia, classified as non-governmental organisations and charities, contracted by District Health Boards, private providers, or Māori and Pacific providers (see Additional File 1). Thirty organisations meeting the eligibility criteria were identified though Google searches across Aotearoa New Zealand. We purposively selected and approached national organisations *N* = 6, Māori providers *N* = 13, Pacific providers *N* = 11, rural providers *N* = 5 and local or regional dementia services *N* = 4 via email and phone call. The response rate was 66.6%.

**Fig. 2:**
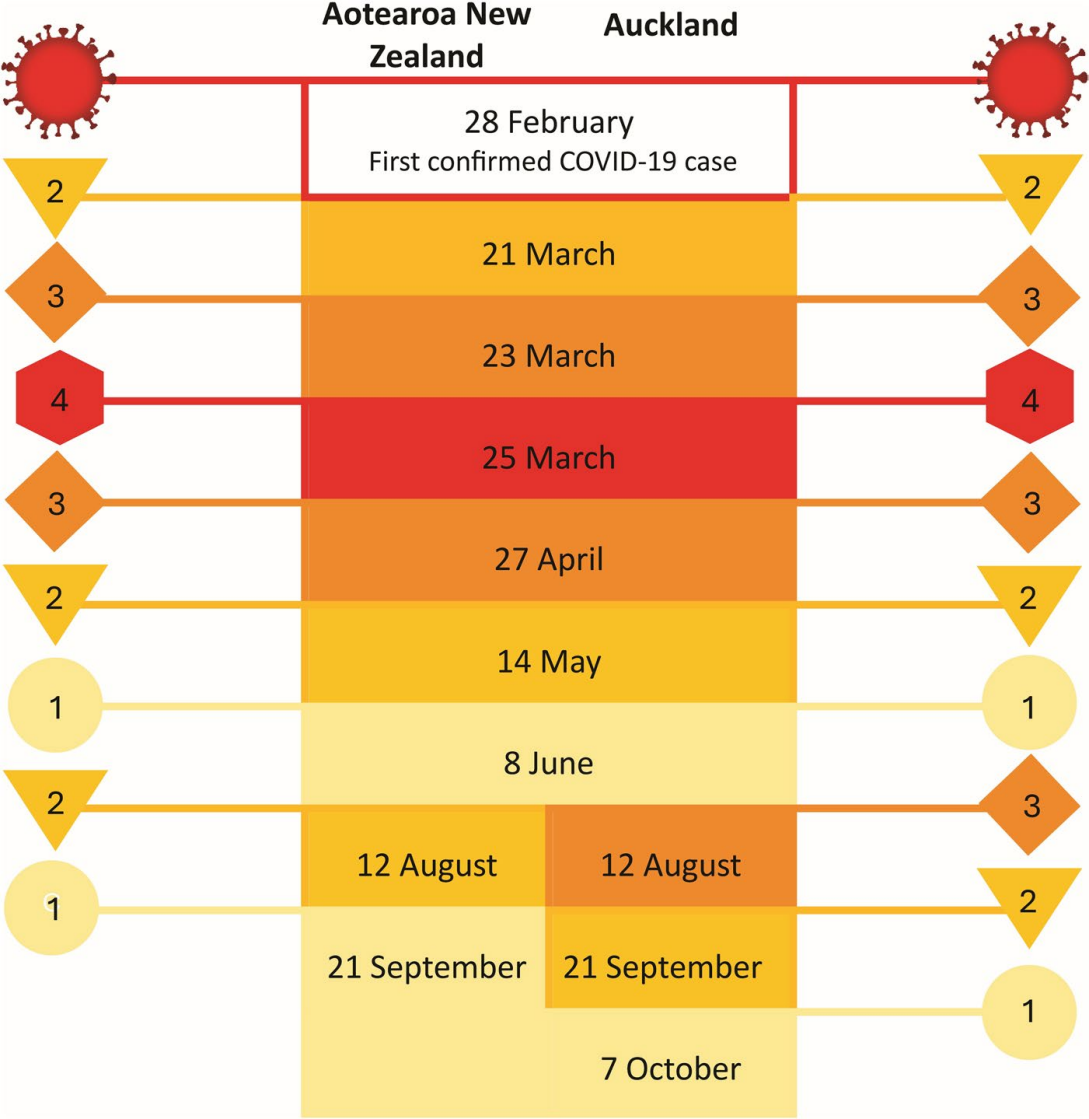
2020 Timeline for COVID-19 alert levels in Aotearoa New Zealand and Auckland Region

Recruitment of service providers (SPs) and data collection took place between 7th July and 4th October 2020, during which time regions in Aotearoa New Zealand moved between four Alert Levels (Fig. 2) described in Table 1. Throughout alert levels, healthcare providers were required to adhere to stringent infection prevention and control measures, including the use of PPE, physical distancing, and hygiene practices to protect both patients and healthcare workers.

### Procedures for data collection

Our research team comprised three social scientists with PhDs and expertise in gerontology (VB), human geography (JW), and health and organisational communication; three nursing researchers with PhDs and specialties in physiotherapy, palliative care and spirituality; Māori and Pacific community researchers; and research assistants. The principal investigator (VB, female) and a research fellow (female) conducted telephone interviews. Three of the authors (VB, JW and AS) contributed to analysis of the data, and reporting findings.

The semi-structured interview guides were developed by the research team [14] and designed to glean information that would correspond to each level of the critical ecological model (Additional File 2). Telephone interviews lasted 30–45 min. SPs were asked about the purpose of the organisation, services they delivered prior to the pandemic, and the development of new protocols and innovative practices during the pandemic. We enquired about challenges faced by SPs, community level influences on the distribution of services, and whether national coordination of services and resources met organisational needs. SPs were also asked about their perceptions of caregivers' unmet needs, but these data are not used in this article.

### Analysis

**Fig. 3:**
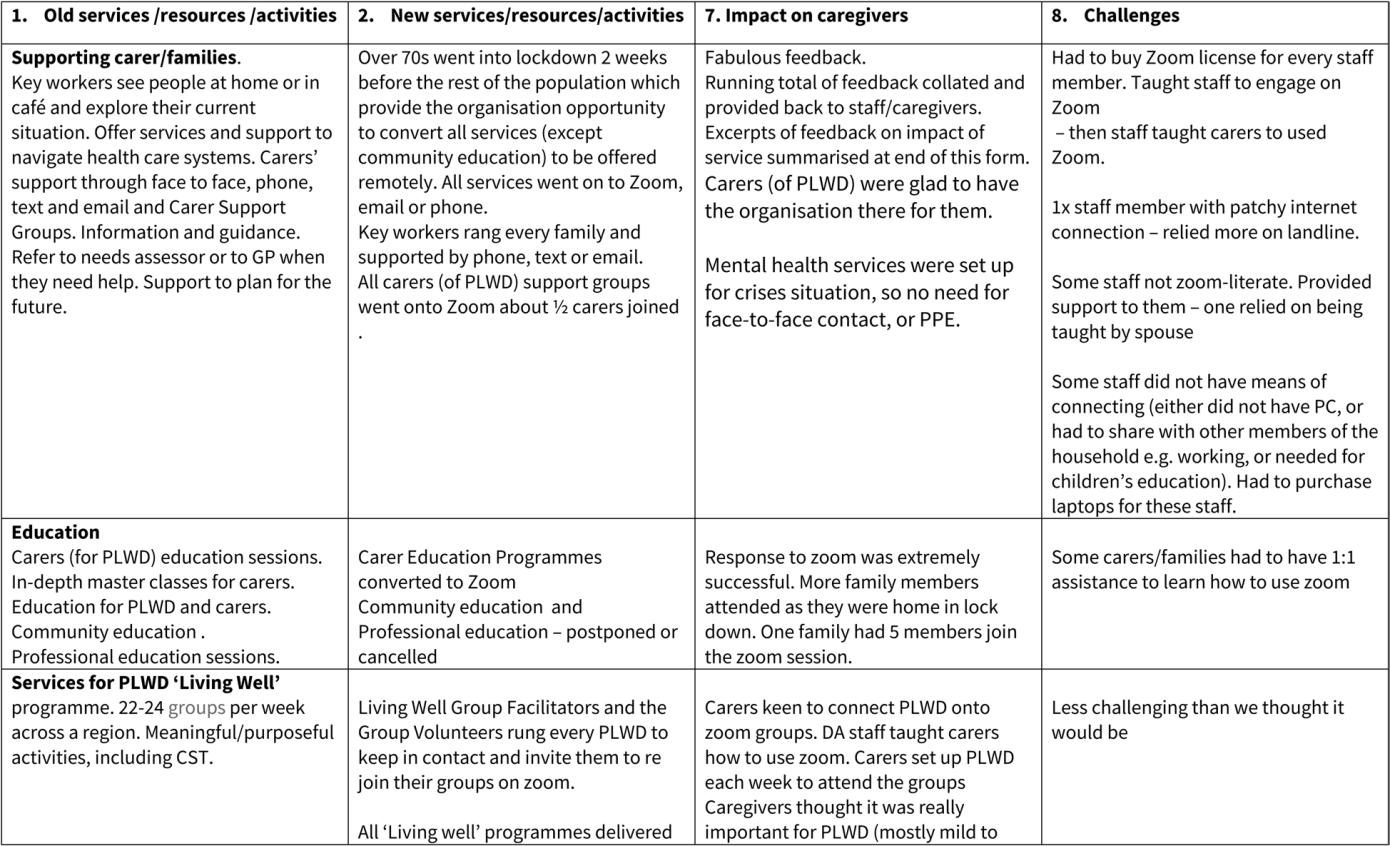
One-page example of the rapid appraisal procedure grid used during interviews

The study was designed to provide rapid data analysis to inform evidence-based public health responses during the first wave of the pandemic, and to minimise the burden for research participants who were struggling with the delivery of health care responses during lockdown [23]. Thus, interviews were not recorded and transcribed. Instead, notes were taken during the interview on a grid with column headings following the structure of the interview topic guide, supplemented by immediate post-interview reflective journaling, and revision of the notes into a coherent summary [24]. Rapid Appraisal Procedures (RAP) grids are commonly used in rapid qualitative research to summarise emerging findings [25]. Participants were sent the revised RAP grid to verify and/or amend and to return to the research team within one week along with examples of innovative resources mentioned during the interview [26, 27] (Fig. 3).

Data were analysed using a hybrid deductive-inductive thematic analysis. NVivo 14 software facilitated rapid deductive thematic coding aligned to an a priori framework based on levels of the critical ecological model and sections of the interview (see Code Derivation in Table 2). Inductive subordinate themes identified within the data were coded within the ordinate deductive themes. VB coded the data which was validated by JW. No new themes were identified beyond coding the fifteenth RAP grid, indicating that data saturation was reached. A summary was presented to an Advisory Group comprising community group leaders and scientific advisors, who corroborated the authors’ interpretations and provided external confirmation that data saturation was achieved.

**Table 2:** Thematic coding structure

Codes	Code Derivation
Social structural and cultural level	Deductive Sect. 5
Government support to SP	Deductive Q18
Government and regional coordination of services	Deductive Q17
Designation of services	Inductive
Structural support and services that worked well	Deductive Q19
Organisational level	Deductive Sects. 1, 3
SP new operating procedures	Deductive Q8
Organisational collaboration	Deductive Q17
Challenges with delivering support by SPs	Deductive Q8 Q15
Community level	Deductive Sect. 3
Availability and suitability of facilities and services	Inductive
Interpersonal level	Deductive Sects. 1, 5
SP relationships and communication with clients	Deductive Q8 Q15
Digital access	Inductive
SP changes in care and support during COVID19	Deductive Q6
In person	Inductive
Informational support	Inductive
Emotional and practical support	Inductive
New assessment, referral and crises	Inductive
Remote working	Inductive
Informational support	Inductive
Emotional and practical support	Inductive
New assessment, referral and crises	Inductive
Arts, leisure, entertainments and culture	Inductive
Checking in and checking up	Inductive
Individual level	
Challenges with workforce or employees	Inductive

## Results

Table 2 shows the thematic codes used in this article. Following a description of the participants’ characteristics, the results are presented under headings representing different levels of the critical ecological model, that is, public policy and culture, organisations, and interpersonal. Themes relating to challenges (i.e. with delivering support, and workforce or employees) digital access, and the availability and suitability of facilities and services, are presented in separate section as they pertain to interactions between levels of the model.

### Participants’ characteristics

Twenty SP CEOs and their nominated representatives were interviewed: National organisations (4); Māori providers (3); Pacific providers (5); rural providers (4); and local or regional providers focusing on dementia (4). Participants had been in post from 0–21 years (Mean 7.37 SD 6.56).

### Policy and culture

Policy and culture are addressed in the themes ‘Government Support and Structural Support’ and ‘Services that Worked Well’, which are presented together. A separate section addresses ‘Government and Regional Coordination of Services’. The theme ‘Designation of Services’ is addressed under the theme ‘Organisations’, and ‘Identifying Vulnerable People’ is addressed under the separate section addressing challenges as both illustrate interactions between different levels of the model.

#### Government support and what worked well

Live daily COVID-19 briefings were presented by the Prime Minister and Director-General of Health, beginning in March 2020. The briefings were a key source of information support for the SPs; the frequency of briefings became more sporadic after June 2020, when the pandemic situation stabilised.

Informational support, financial and practical support provided by the government, and Ministries of Social Development, Health, and Business, Innovation and Employment enabled organisational resilience for SPs. Co-ordination of services between SPs was managed by sixteen Civil Defence Emergency Management (CDEM) Groups, each comprising committees of elected councillors from within regional boundaries working in partnership with emergency services, lifeline utilities, District Health Boards, health and care SPs, and government departments.

Some government ministries played specific roles supporting SPs. The Ministry of Social Development provided wage subsidies for staff and honoured contracts although many SPs could not meet service targets while face-to-face contact was prohibited. This ministry also funded service innovations for clients proposed by SPs (e.g. food packages or digital technology). SPs stated that the funding from the Ministry of Social Development was easy to apply for and funds were obtained rapidly.

The Ministry of Health provided funding for COVID-19 specific services, and helped SPs develop policies fulfilling the Health and Safety at Work Act 2015 to mitigate risk and protect employees from workplace hazards. National SPs were in regular direct contact with the Ministries of Health or Social Development (depending on the focus of their services) for briefings and updates. Information from these meetings was cascaded down to staff within organisations, and experiences of frontline workers and local SPs were passed up to the Ministries.

The Ministry of Business, Innovation and Employment provided extensive resources, templates, and guidance documents to help SPs implement human resource policies, such as risk assessment, health monitoring, flexible working arrangements, leave and absences management, employee wellbeing and support (e.g., Employee Assistance Program), and wage subsidy schemes. The goal was to ensure that workplaces remained safe, employees were supported, and SPs could navigate the operational challenges posed by the pandemic. The Māori Health Directorate, Pacific Future Fund and Ministry of Health provided funding for innovative services and activities targeting Māori and Pacific communities.

#### Government and regional coordination of services

CDEM worked in collaboration with other government agencies and SPs to identify ‘vulnerable’ individuals in need of additional support to include on the Civil Defence list (see ‘challenges’ below). Vulnerability was considered on basis of age, health conditions, disability, or other factors that made it difficult to manage independently. People on the Civil Defence list were prioritised for receiving essential services, such as grocery deliveries, prescription medication, and ongoing welfare checks. In practice, SPs identified ‘vulnerable’ people, and provided the essential services. One Pasifika SP noted although the Civil Defence provided a list of SPs, it was still difficult to “decipher who was doing what in the community – finding who had the right support for a particular situation” (SP04). This sentiment was echoed by others.

Locally, there were differences in communication, leadership and coordination of services by District Health Boards (DHBs). One SP noted DHB leadership was absent at the pandemic outset, another SP did not receive any direct contact despite sending summaries of their activities. Yet there was evidence from several SPs of good communication and support from DHBs. One SP said,

*“the support our DHB has offered us cannot be faulted, when we went into Level 3 lockdown our day program services were in question, and the viability of the organisation looked very uncertain. The quick and decisive decisions made enabled us to keep all of our staff engaged and fully paid.”* (SP07)

Distribution of PPE to frontline healthcare workers, hospitals, and other essential services was co-ordinated by the Ministry of Health. Centralised distribution ensured that PPE was allocated based on priority needs. While this worked for some organisations, other SPs encountered difficulties. Several had to lobby the Ministry of Health directly, or via the Ministry of Social Development to get PPE released to them.

### Organisations

Service providers’ procedures and strategies were influenced by the designation of their services as either essential or non-essential and these themes are presented together. ‘Organisational Collaboration’ is also addressed in this section.

#### New procedures and strategies and designation of services

The essential services regime allowed some premises to keep operating during COVID-19 (see Table 1). In March 2020 essential services included District Health Boards and their facilities; any person employed or contracted as a doctor, nurse, pharmacist, paramedic, kaiāwhina,^1^ social worker, aged (residential and nursing) care and community workers and paid caregivers more generally; hospitals, primary care clinics, pharmacies, and care facilities. Only organisations deemed ‘essential services’ were able to continue face-to-face services with clients, with the appropriate PPE protocols in place. The classification of essential services was adapted when the country entered Alert Level 4 for the second time in August 2020 to include social and community-based services supporting persons to maintain critical well-being.

There were variations in approach by national SP organisations versus local organisations serving smaller or specific populations. Although the preparation of emergency plans had been devolved to local organisations, during the pandemic it became apparent that these had not always been completed. Subsequently, national SPs developed emergency plans and distributed these to local organisations.

The national SP essential workforce required new health and safety training, but as one noted “with everyone in lockdown we needed to completely change the way we trained our people” (SP11). For example, training teams created videos on “donning and doffing” (how to put on and take off PPE) to be utilised by all staff. One national SP delivered PPE to letterboxes of staff and/or clients; others adapted everyday operations, limiting the need for staff to enter clients’ homes by developing a triage process, or delivering care differently to enable physical distancing whenever possible.

Digital applications were used more extensively, for example, to provide client information, alert to additional risks (e.g., an unwell client), and co-ordinate visits. Two national SPs implemented telehealth procedures, checklists, or standard questions to assist teams assessing clients’ issues.

Development of new strategies and procedures by national SPs was not universal. One local SP noted ‘disappointment’ in the leadership of their national organisation, feeling that the local provider was “left to fend for itself” (SP05).

In contrast to national SPs, the strategic response of local SPs tended to focus on meeting clients’ most pressing needs with a dwindling workforce (see ‘challenges’ below). Some changed the focus of their work. For example, one SP switched to providing personal care only, dropping housework; as they noted, “clients can live with mess, but not without being washed” (SP16). A rural SP suspended a policy prohibiting involvement in financial transactions to help clients obtain medications or groceries during Level 4.

A Pacific SP which was designated as a non-essential service during the first Level 4 put all other work on hold to manage an 0800 number to help clients complete welfare forms online, which could be used to apply for support (e.g., Wage Subsidy Scheme; COVID-19 Leave Support Scheme; Emergency Benefit; Rent and Housing Support; Food and Essential Services Support; and Health and Disability Support).

#### Organisational collaboration

There were examples of collaboration between organisations at the national and local level. For example, the Chief Executives of national SPs collaborated to share information. Extant and new collaborations within Districts also facilitated coordination of services and information. One SP noted strong relationships with other health and care providers in the locality assisted with planning responses to restrictions and solving problems.

### Interpersonal

Innovative practices for interaction were developed and delivered remotely or in person. In this section we combine and compare in person and remote ‘Informational Support’, ‘Emotional and Practical Support’, and ‘New assessment, Referral or Crises’. ‘Checking In and Checking Up’, and ‘Arts, Leisure, Entertainment and Culture’ were only delivered remotely.

#### Informational support

SPs were often seen by clients as good sources of information. For example, one noted, “we established an 0800 number so that older people and caregivers could phone up for trusted information, as digital literacy and navigation of multiple sources of information could be difficult.” (SP07).

Remote informational support included material about COVID-19 or the protocols relating to social restrictions at each Alert level, and the use of PPE. Four SPs made information available in appropriate and accessible formats for their clients (e.g. large font size, audio, or translated). Nine SPs provided information on accessing health and care support, or advice on how to maintain mental, physical and spiritual health, keep connected, and support a person at home. Two SPs provided practical support by providing digital devices, with instructions.

There were examples of in-person information support, such as, support workers delivering information about COVID-19 to clients. In-person informational support was particularly important in Pasifika communities where one SP trained volunteers to deliver information in various Pacific languages.

#### Practical and emotional support

In-person practical support included financial support (e.g. food vouchers, energy bills, broadband plans, pre-paid phone cards), food parcel delivery, digital devices such as tablets, mobile phones and laptops, and care packs. There was also practical support for those who could not go out, such as pharmacy or medication pickups, and grocery shopping and delivery.

Often practical support was tailored to a particular group. For example, one SP produced a new ‘companion’ card for unpaid caregivers to use when in public, explaining the caregiver had to be accompanied by the care recipient with dementia. A Māori SP provided care-packs including essentials required to keep people well in overcrowded houses. Both Māori and Pacific SPs paid electricity bills and delivered wood for fires (for heating and cooking), along with food parcels and food vouchers as many of their clients could not afford to pay bills.

SPs also devised ways of providing in-person emotional support to unpaid caregivers, for example through driveway visits, chats through windows or on porches, and a staff choir that sang outside clients’ homes.

While some SPs had to suspend face-to-face support to clients, service delivery was adapted, and practical and emotional support was delivered by phone, email, and video calls. This included online/video conference caregiver support groups and for people living with dementia, and exercise classes. One SP delivered Cognitive Stimulation Therapy to people with dementia via online video link [28], while another SP set up a Facebook page to enable clients who used an Individualised Funding model to share their experiences.

#### New assessments, referrals and crises

New assessments were mostly conducted remotely. In the first Level 4, between March 25th to June 30th, 2020, the interRAI Contact Assessment was managed over the phone as a rapid (approximately 20 min) temporary alternative to the interRAI-HC assessment. SPs described how more triage was undertaken over the phone; a Registered Nurse would carry out a medication review and the organisation would liaise with the GP and pharmacy.

In anticipation of carer crises, some national SPs developed emergency plans for unpaid caregivers to complete. This provided a written record of preferences and needs of care recipients, if the caregiver became ill (or passed away).

In-person support was mostly provided for crises and emergencies. For example, SPs referred care recipients to emergency home-based or relocated to residential care when caregivers passed away during restrictions. As part of driveway chats (see above) SPs also undertook ‘welfare checks’ and referred clients on to hospital-based services or GPs if required. One SP transported clients to medical appointments wearing PPE.

#### Arts, leisure, entertainment and culture

Many SP were concerned that disruption to routines and resources like support groups would be detrimental to their clients, and unpaid caregivers would struggle to find things to do. In response eight SPs developed entertainment resources delivered by email or hosted online. For example, one SP sent a daily newsletter to unpaid caregivers, and links to virtual activities (e.g. art gallery tours or train journeys) for people with dementia. One SP collaborated with experts to deliver online art groups and themed meetings, while others created their own arts and crafts classes or distributed digital devices with preloaded games.

Due to lack of access to and difficulty using digital devices among people with dementia, one SP created and hand-delivered 300 activity booklets. Other SPs developed, delivered or mailed hard copies of entertainment resources, tailoring them to the severity of dementia or cultural context (i.e. for Māori and Pasifika clients). While most of the ‘hard copy’ entertainment packs were developed for unpaid caregivers of people with dementia, two SPs delivered hard copies of entertainment activities to the homes of clients who did not have email.

#### Checking in and checking up

As most face-to-face services were moved to contactless services, ‘checking in’ became a virtual care modality and provided a sense that ‘someone was there’. On the other hand, ‘checking up’ referred to contact intended to ascertain the wellbeing of clients.

Many SPs were restricted to remote contact only, especially in the first lockdown. Thirteen participants talked about the strategies they adopted to maintain communication with clients, ranging from regular telephone calls, texts, emails, or video calls to instant message or group conversations. Two SPs focused on establishing how much contact clients would like so that they were neither isolated, nor overwhelmed. SPs felt it was important to “be a voice on the end of the phone, giving re-assurances and connecting people with other services” (SP13). Some SPs relied on telephone calls only, others tried to ascertain the status of digital connectivity and whether clients could receive emails.

SPs perceived that ‘checking in’ was particularly important for some client groups. For example, to help maintain routines for unpaid caregivers supporting a person with dementia, one SP phoned on the day that they would normally go to a day programme. Phone calls to older Pasifika people and caregivers were also perceived as essential as verbal communication was considered much better than leaflets.

Regarding ‘checking up’, twelve SPs initiated phone calls to all their clients for wellbeing checks, while another received a request from a GP practice to call all their clients over 70. One SP noted that support workers were worried about clients and would often take it upon themselves to check up on them in their own (unpaid) time. Another SP noted there were more welfare checks during COVID-19 than prior to the pandemic.

### Challenges

The challenges identified by SPs occured at different levels of the critical ecological model: organisational challenges related to support delivery, community challenges concerned service availability and suitability, interpersonal challenges involved digital access, and individual challenges were tied to workforce resources and characteristics. However, each theme illustrates interactions between systems within the critical ecological model.

#### Challenges with delivering support

At the public policy level, CDEM groups created a list of ‘vulnerable adults’ that were prioritised to receive services during the pandemic, based on age, health conditions (e.g. cardiovascular disease, respiratory issues, diabetes, and cancer), disability (e.g. physical, sensory, intellectual, or mental health disabilities limiting ability to access essential services or comply with public health measures), and social isolation, dependence on services, ethnic and cultural factors (e.g. Māori and Pasifika communities). However, at the organisational level, the list relied on the identification of vulnerable adults by SPs and the combination of criteria used varied significantly. For example, one SP prioritised individuals living alone with dementia, another focused on those over 85 with high-level needs. Another categorised clients as home alone, living with spouse, or living with others, and considered their access to service, transport, health of the care recipient and their primary caregiver, geographical location, and digital access. Factors like social isolation, disability, and economic hardship were weighted variably (or not considered) by other SPs. 

Identifying vulnerable people relied on the SPs’ community connections and knowledge of clients prior to COVID-19. Furthermore, there was little or no familiarity with people who developed a need for care after the onset of the pandemic. Unless unpaid caregivers proactively contacted SPs, they were in danger of being overlooked for inclusion on the Civil Defence priority list. As criteria were not always applied consistently to old and new clients, the ‘type’ of older people and caregivers receiving home-based services varied across different SPs and regions.

#### Availability and suitability of facilities and services

At the community level, SPs identified a challenge relating to clients’ changing circumstances during the pandemic, particularly the need to access aged residential care (ARC) when a caregiver was no longer able to provide support at home, needed respite from caring, or in emergencies. This was extremely difficult during Alert Level 4, when the most stringent restrictions were in place and demonstrates the interaction between policy and community levels of the critical ecological model. One SP noted that after a caregiver passed away, emergency ARC was required for a person living with dementia. The SP was ‘literally kicking the door’ as staff would not admit them. The SP threatened to phone the Human Rights Commissioner before the issue was resolved. Another SP noted that rest homes would not accept new residents to provide caregivers with respite without following rules that were often unacceptable to the caregiver and care recipient.^2^

#### Digital access

At the interpersonal level of the model, digital access was a challenge for both staff and clients of SPs. There was great variation in digital literacy within both groups. Digital access exposed inequalities that had not been sufficiently addressed at the policy level, such as inadequate infrastructure, digital literacy and limited access to equipment.

During levels 3 and 4 when many staff worked at home, SPs had to upskill their workforces in relation to using digital equipment, apps, and systems. Many SPs delivered training in telehealth, video conferencing, and using the range of smartphone features to their staff.

SPs noted some staff were either not interested or unwilling to use digital technology, or there was competition between household members to use digital devices at home. Other SPs did not have appropriate digital hardware or software for staff. This was especially relevant in organisations which employed older staff or relied on older volunteers, many of whom did not have an internet connection, laptops, computers or printers at home, or else knew how to use mobile phones only for calling or texting. While essential services were able to purchase laptops for staff who needed them, SPs designated as non-essential services did not receive funding to rectify this. Consequently, where possible, digitally literate staff used their own personal computers and phones for work purposes, while others used telephone calls as the main form of communication.

The proportion of clients with digital access (and digital literacy) also varied between organisations. SPs estimates of the proportion of their clients that had an email address ranged from 30 to 90%. Some SPs adopted a policy to connect to clients by phone, others were unable to support those who were not connected digitally.

Digital access was particularly difficult for certain groups such as clients in rural areas, people living with dementia, and older Pasifika. One SP noted difficulties for clients accessing online resources because of lack of rural network coverage; while a SP working with people living with dementia noted that around 40% of clients lived alone, and most only used a landline. Cognitive impairment precluded some people with dementia from using digital devices. Another SP noted that Pacific older people without phones or who forgot to charge phones were difficult to contact.

Some SPs attempted to address digital inequities by providing training and resources. One SP provided training for unpaid caregivers to use video conferencing, another delivered a computer course for older people during Alert Level 1 and 2 (June to Aug 2020). The training and distribution of laptops before Alert level 3 restrictions in August 2020 facilitated better communication with clients during lockdown.

#### Challenges with workforce or employees

During the pandemic the SP workforce shrank, with more than 30 per cent of staff on unplanned leave at any one time. Staff were unable to work if they were unwell or had been exposed to COVID-19. SPs noted others were educating children at home which impacted on their ability to work, and some took leave to manage burnout, exhaustion, and grief.

Social restrictions designed to reduce the risk of contracting COVID-19 for particular groups also impacted the workforce. For example, older volunteers were asked to step down as they were unable to attend face-to-face activities or deliver ‘meals on wheels’. One rural SP noted many of their support workers had respiratory issues that were attributed to coal smoke in the town, demonstrating an interaction between the community and individual levels of the model.

SPs noted staff were worried about transmission of COVID-19 between clients’ homes and their private abode. Consequently, some support workers were unwilling to extend their bubble and would only work with existing clients. Others, especially Māori SPs, faced challenges recruiting new staff to fill vacant roles, attributing this to the low status and undervaluing of homecare work.

SPs also noted challenges with travelling across regional boundaries to deliver home-based health and care services to clients when alert levels varied by region. Essential workers were provided with contradictory advice about moving across boundaries and there were inconsistencies between agencies involved in the policing of regional borders.

## Discussion

Establishing best management practices in country-level health system responses is vital to improve national preparedness to mitigate the effects of other future unexampled events. A critical ecological model is useful for understanding absorptive, adaptive, and transformative health system resilience because it examines multiple, interconnected levels—individual, interpersonal, community, organisational, and societal. By analysing health system resilience through this lens, we can identify how factors at each level contribute to a system’s capacity to absorb shocks (absorptive resilience), adjust to changing conditions (adaptive resilience), and fundamentally transform in response to crises (transformative resilience). In exploring absorptive, adaptive and transformative health system resilience [11] we start by discussing positive contributions observed in our results and move on to consider gaps and challenges undermining resilience. We contextualize our findings by comparing and contrasting them with existing international literature.

At the policy level, Aotearoa New Zealand government acted quickly and decisively with strict lockdown measures, clear communication strategies, activating emergency response plans and designating essential workers [29]. Internationally, similar measures have been acknowledged as characterising effective health system responses to COVID-19 [30] These were examples of absorptive resilience, withstanding and managing the immediate shocks of COVID-19 without major changes to structure or functioning. However, most responses to the pandemic in Aotearoa New Zealand provided examples of adaptive resilience: adjusting and reorganising responses to the changing conditions.

The government adapted to the new situation by providing support and protection for healthcare workers [31]. This included good governance, the provision of PPE, and financial aid to individuals and businesses. This was not observed universally as gaps in the PPE supply chain disrupted provision to health care workers in other countries [32]. Financial aid mitigated some of the economic impact of the pandemic for financially vulnerable older adults and caregivers, and helped preserve jobs, especially in the community care sector in Aotearoa New Zealand with similar actions taken in other countries (e.g. Australia, Austria, Wales, Scotland) [33].

At the organisational level, SPs modified service protocols, enhanced telemedicine services, and redistributed resources to vulnerable people by remote or face-to-face means. This was facilitated by rapid adoption of digital health solutions and temporary fixes, including telehealth services and apps that helped with rapid information about clients and coordination of care workers. Because COVID made traditional ways of working unviable, digital solutions were rapidly adopted by community services globally [34]. SPs led community initiatives to support vulnerable populations, ensuring access to essential services and reducing social isolation. Active involvement and leadership from Māori, Pasifika and rural communities ensured culturally appropriate responses and better outreach to these populations. In contrast to the Regional Health Boards’ response in Canada, where 13 out of 16 Northern and Indigenous regions offered public health messaging translated into Indigenous languages and guidance on participating in traditional practices [35], the approach in Aotearoa New Zealand relied heavily on the provision of information through SPs.

These positive aspects of health system resilience highlight the importance of leadership, community involvement, robust communications systems, good data management, collective and individual trust, and a well-prepared healthcare system in managing public health crises. Despite these positive aspects, there were also gaps identified. Our research highlights challenges at each level of the critical ecological model and limitations in the health system, such as inconsistencies in the identification of vulnerable individuals, coordination challenges, communication breakdown, staffing issues, and geographic and socio-economic barriers.

Co-ordination of services between SPs and the identification of ‘vulnerable people’ was managed by the CDEM groups. However, coordination between multiple organisations was often difficult, leading to delays or gaps in service delivery. Although the priority list of vulnerable people aided the distribution of support to some, there was lack of a unified framework or standard guidelines for identifying and prioritising vulnerable populations. Health agencies primarily focused on medical vulnerability, while social service organisations placed greater emphasis on economic and social factors. Māori and Pasifika organisations, as well as other community groups, often had their own criteria for vulnerability, reflecting the unique needs and circumstances of their populations. Ability to identify individuals depended on the level of information held by organisations prior to the pandemic. As a result, SPs used varying criteria to identify vulnerable individuals, leading to inconsistencies and some people being overlooked. New service users faced additional barriers to accessing services if they were not already known to SPs, especially if they were unable to identify which SPs they should contact. Identifying and supporting the most vulnerable older adults during the pandemic has also been identified by SPs in Canada as a priority for future health system resilience [36]. Enhanced coordination of emergency planning alongside the development a country-level unified framework to inform a ‘priority list’ may address these concerns.

SPs identified other barriers to delivering services at the community level. Some regions or communities had better resources and networks, while others were disadvantaged by border controls, resulting in uneven distribution of support. This meant that inequities in access to services were amplified for those in rural or remote areas [37] as people often faced greater challenges in receiving timely support. Māori and Pacific SPs reported on developing innovative services for their communities. However, these were typically delivered in areas with high proportions of specific cultural population. For Māori, Pasifika, and migrants living in less intensive concentrations of culturally distinct groups, cultural and language barriers may have impeded effective outreach and support [38, 39].

At the interpersonal level, in addition to language difficulties, communication breakdowns between the government and society were often due to a preference or need for face-to-face information or digital access. Some older people and unpaid caregivers did not have had access to reliable communication channels (e.g., phone or internet) to request help or receive updates. Some SPs tried to remedy this by providing digital training, equipment, or ‘data’ (i.e., prepaid vouchers or plans) to unpaid caregivers and older people. Other studies have noted that there were barriers to implementing eHealth during the pandemic, especially in rural areas. However, local service providers quickly developed their own competencies and supported clients in acquiring similar skills [40]. While the government in Aotearoa New Zealand recognised a digital divide in certain segments of society [41, 42], there was little attention given to the limited digital literacy and access experienced by many health and community care staff, and the subsequent workforce challenges encountered by SPs. To enable effective communication and service delivery in future emergences, digital exclusion should be addressed through transformations within the health system and society.

Staffing issues were apparent across most SPs, and were also noted as a factor influencing the supply of services in 29% of countries surveyed by WHO in 2020 [29]. In Aotearoa New Zealand, some staff shortages were attributed to their digital access and digital literacy influencing their ability to work from home, and the age of some employees and volunteers. Staff also faced additional caring or educational responsibilities, illness, and increased stress, or were safeguarding family; similar issues for front-line community health and social care staff have also been reported elsewhere [43–45]. Most SPs reported being overstretched during the pandemic and, some initiated triage systems to reduce pressures on staff (also tried elsewhere [46, 47]). Most community care workers experienced both increases and changes to their workload due to staff shortages, which has also been documented internationally [29, 44, 47, 48].

Difficulties recruiting staff to replace the depleted critical workforce of health and care community staff were reported in this study and elsewhere [49]. Some SPs provided digital training and equipment for staff while others lacked the financial resources or knowledge to do this. Other countries facing staff shortages employed different tactics such as recalling inactive health and care workers, or fast-tracking trainees to provide childcare for essential health care workers [31]. These are important strategies for health care systems to develop a broader capability and draw on a wider pool of potential staff in anticipation of future emergencies [50].

Some of the issues identified in this study, such as workforce sustainability and digital access, were systemic issues that were not addressed sufficiently before the pandemic. In 2020, prior to the pandemic, the health and disability system already experienced serious issues, with a workforce under pressure and high stress levels [50]. Community care workers were overlooked as ‘essential workers’ during the first Alert Level 4 lockdown, and PPE provisions for this sector were neglected. Similarly, today, strategies for recruitment and retention, and training for staff development in areas such as digital and data literacy focus on health professionals rather than the broader community care sector [51]. For transformative health system resilience, new strategies are required to ensure adequate workforce capability, capacity, and support in future emergences.

Other studies have also highlighted how inadequate health workforce diversity, insufficient training and remuneration, and limited support and protection weaken health system resilience to public health threats. These factors reduce the capacity of health systems to maintain equitable primary health and community service delivery while simultaneously addressing the demands of health emergencies [52]. In many countries, the community care sector has been pushed to breaking point and has not recovered from the effects of the pandemic on the workforce [53–55]. Without transformational work in these settings, the community care sector will continue to lack resilience, and its precarious situation means that health systems will be unable to deal adequately with future emergencies [56].

The study has some limitations. The study was conducted during the early stages of the pandemic (July to October 2020) with only 20 SPs, so it may not reflect the ongoing or long-term challenges faced in later waves of COVID-19 or fully capture the experiences of all providers across the country. Furthermore, the study was conducted in a specific political, cultural and geographical context, which may influence the findings. The challenges might vary significantly in different healthcare systems or cultural settings, limiting the applicability of these results outside of Aotearoa New Zealand. A key strength of this article lies in its focus on the resilience of primary health and community services to support unpaid caregivers and older people within the broader health system which is lacking elsewhere [30]. By examining the resilience of primary health and community SPs, the findings contribute valuable insights into the overall resilience of the health system, enhancing the understanding of its capacity to withstand and adapt to challenges. The challenges faced in Aotearoa New Zealand are not unique, but have also been encountered in other countries, suggesting broader implications for global health system resilience.

Consistent with findings from other studies conducted during the pandemic [30], our research primarily highlights aspects of absorptive and adaptive resilience. Transformative health system resilience can only be achieved by leveraging lessons learned from the COVID-19 crisis as a catalyst for change. Our investigation into the influences of different levels of the critical ecological model on the resilience of primary health and community SPs contributes to this endeavour. For health systems that encountered challenges like those observed in the Aotearoa New Zealand context, we offer the following recommendations.

- **A Unified Framework to Inform a ‘Priority List’**: develop national standards for identifying and supporting current (e.g., through administrative datasets) and new vulnerable individuals, ensuring consistency across regions and organisations
- **Adequate Workforce Capability, Capacity, and Support**: strategies for recruitment, retention of community care workforce, and ‘diversity’ in the workplace (e.g., a reserve pool of inactive workers) so that in the event of disasters there are people that can step-in and help.
- **Enhanced Coordination of Emergency Planning**: establish clear roles and responsibilities for all SPs to avoid duplication of effort; and ensure SPs have local emergency response plans, are digitally enabled (i.e., have sufficient resources to facilitate home-based working in times of emergency), and have access to emergency facilities (such as residential care facilities and hospitals).
- **Effective Communication:** address digital exclusion of older people, caregivers and staff within service provider organisations; develop multi-channel communication strategies to reach all segments of the population, including translations from a single or central point to avoid misinformation concerning complex concepts and instructions.

## Conclusions

While Aotearoa New Zealand is justifiably considered to have done well in preventing the worst impacts of Covid-19, there are lessons that can be learned about health system resilience. This unexampled event provided challenges for the health system and there were good examples of absorptive and adaptive health system resilience in the actions of SPs and Government responses The government demonstrated absorptive resilience through swift actions, including lockdowns and clear communication, while adaptive resilience was shown by adjusting support measures for healthcare workers and vulnerable populations. SPs played a vital role in modifying service protocols, enhancing telemedicine, and redistributing resources, demonstrating adaptive resilience. Knowledge gleaned from these observations of absorptive and adaptive resilience alongside the barriers and challenges to resilience could contribute to Transformative Resilience. This requires long-term strategic changes to enhanced coordination of emergency planning, such as developing a unified framework to inform a ‘Priority List’, strategies to ensure adequate workforce capability, capacity, and to support effective communication by addressing digital exclusion. These steps could address vulnerabilities and enhance capacity to improve the health system robustness and sustainability in the future, especially regarding preparedness for future crises.

## Supplementary Information

Additional file 1.

Additional file 2.

## Data Availability

The datasets generated and/or analysed during the current study are not publicly available as restrictions apply to the availability of these data due to sensitivity (i.e. Māori data sovereignty). These restrictions have been ratified by the Auckland Health Research Ethics Committee (AHREC) at the University of Auckland. Data will be made available from AHREC (ahrec@auckland.ac.nz) on reasonable request.

## Abbreviations

ARC: Aged Residential Care
CDEM: Civil Defence Emergency Management
DHB: District Health Board
GP: General Practitioner
MBIE: Ministry of Business, Innovation and Employment
MoH: Ministry of Health
MSD: Ministry of Social Development
PPE: Personal Protective Equipment
SPs: Service Providers

## Footnotes

1 Kaiāwhina describes non-regulated roles in the health and disability sector. Kaiāwhina is a Māori language term that embodies the core essence of a workforce that is passionate, resilient, diverse, skilled and committed to supporting hauora (holistic wellbeing) outcomes.

2 The process involved the person obtaining a clear Polymerase Chain Reaction (PCR) COVID-19 test while isolating at home (separate from the unpaid caregiver) for 48 hours, and then on entering ARC being isolated from others in the facility.
