# Supplementary material for: Understanding the contribution of primary and community services to health system resilience during the COVID19 Pandemic in Aotearoa, New Zealand: a qualitative interview study
Source: BMC Health Serv Res. 2024 Dec 24;24:1650. doi: 10.1186/s12913-024-12078-6 (PMC11667889; doi:10.1186/s12913-024-12078-6)
Supplement: Supplementary file 1 — Additional file 1. [file 12913_2024_12078_MOESM1_ESM.docx]

**ADDITIONAL FILE 1**

## **Study Setting: Aotearoa New Zealand Health System**

The Ministry of Health is responsible for overall policy direction, regulation, and coordination of the health system in Aotearoa New Zealand. Historically, the country was divided into District Health Boards, which managed and delivered health services within specific geographic areas. As of July 2022, the District Health Boards were replaced by a centralized organization known as Health New Zealand (Te Whatu Ora).

At the time of this study, services and support for caregivers was provided through primary care, DHBs funded community services (e.g. Needs Assessment Support Coordination, home help), and non-governmental organisations [1, 2]. interRAI-Home Care (interRAI-HC) - a standardised geriatric assessment tool – is mandated to determine the level of support required by older adults living in the community [3].

Services available to older people living in the community and deemed eligible include home help, personal care, medication management, transportation assistance, social support, or housekeeping, health monitoring and rehabilitation services. Other services include respite care for primary caregivers, and day programs. While many services are free at the point of use (e.g. homecare services), some, such as appointments with General Practitioners (GPs) and prescription medications, require a co-payment. Subsidies are available to reduce these costs for low-income individuals and families, and regions. Long-term care (and respite) is provided in aged residential care (ARC) facilities, which are managed by private companies or non-profit organizations.

The health system aims to provide universal access to essential health services, ensuring that all residents, regardless of income or area of residence, can receive necessary care. The country is founded on Te Tiriti o Waitangi (the Treaty of Waitangi), a contractual relationship between the British Crown and Māori, and includes active protection of equitable health outcomes for Māori. While equity for Māori is embedded in policy, the same focus is not extended to other ethnic groups or by geography [4]. Although the health system has focussed on improving conditions for groups who have had fewer opportunities, health inequities are widening: Māori, Pacific peoples and those with lower socioeconomic status experience much higher levels of chronic disease, higher morbidity and lower life expectancy [4].

**Community Service Provider Classifications**

Community service provider organisations for older people and caregivers in New Zealand can be classified based on their services, governance structures, and funding sources.

**Non-governmental organisations, and charities** are non-profit organisations providing support services such as in-home care, respite care, social connection programs, and advocacy. Organisations may be national, local or community-based organisations. Local and community-based organisations are often smaller than national organisations, and are staffed by volunteers. At all levels, these type of service provider organisations rely on donations, government grants, and charitable funding.

At the time of the study, **District Health Board (DHB) contracted providers** received funding from DHBs to deliver health and social care services for older adults and caregivers. This included home-based support services, nursing care, and caregiver support. They operated within the public health system and often worked alongside other healthcare providers.

**Private Providers** offer paid services, often covering in-home caregiving, personal care, and assisted living arrangements. Clients or their families typically pay for these services directly or through insurance.

**Māori and Pacific providers** are culturally focused organisations providing tailored health and social care services for older Māori and Pacific populations. Services are based on cultural values and traditions, supporting whānau (family) and aiga (extended family) structures. They are funded through a mix of government contracts, community support, and philanthropic contributions.

**Rural Service Provision**

Rural health research in Aotearoa New Zealand draws on many different classifications of rurality [5, 6]. Rural areas can be found within all regions of the country, and often feature small or isolated communities: 20% of the older population aged ≥ 60 years, live in rural areas (using the Geographical Classification for Health) [5]. Service provider organisations that serve rural clients are often located in urban areas, thus complicating defining a ‘rural provider’.

Aotearoa New Zealand is divided into sixteen regions. In this study, the research team’s professional knowledge of rurality (population density and dispersion of settlements) and composition of the population (e.g. comprising both Māori and non-Māori) was used to select specific regions on both North and South Islands from which service provider organisations were selected.

The regions selected for recruitment of rural service providers were **Northland** (population 203,900; population density 15 people per km^2^ [7]; 37.4 % of the population identify as Māori [8]); **Hawke’s Bay** (population 184,800; population density 13 people per km^2^ [7]; 28.6% of the population identify as Māori [8]), **West Coast** (population 32,900; population density 1.4 people per km^2^ [7]; with 13.5% of the population identify as Māori [8]), **Canterbury** (population 666,300; population density 15 people per km^2^ [7]; 10.6 % of the population identify as Māori [8]); and **Southland**: (population 103,900; population density 3.33 people per km^2^ [7]; 16.8 % of the population identify as Māori [8]) (see AF Figure 1).


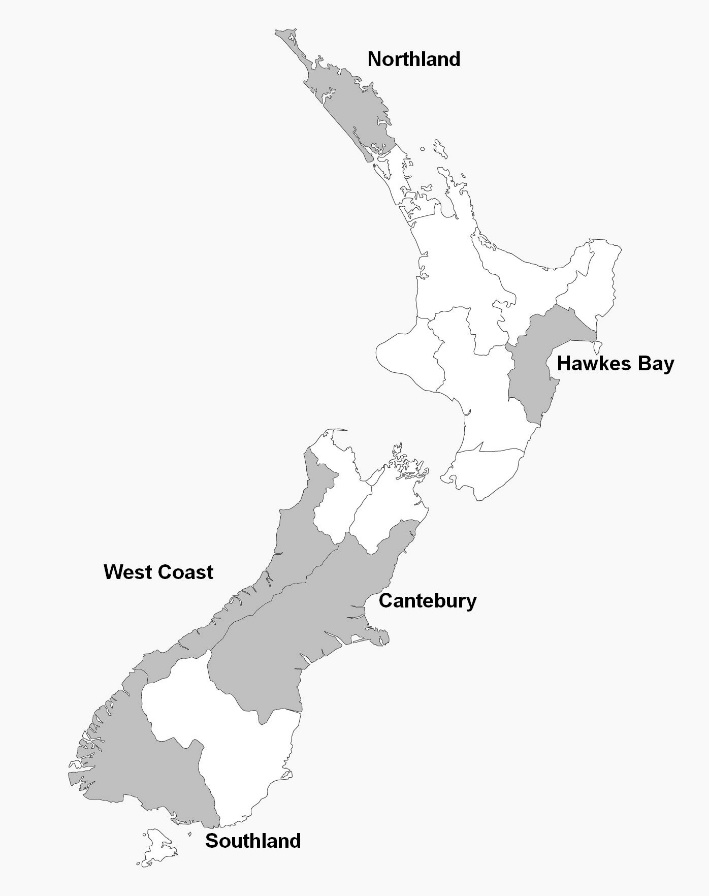
AF. Figure 1. Regions in Aotearoa New Zealand selected for recruitment of rural service providers

[Maps by FreeVectorMaps.com](https://freevectormaps.com/)

**References**

1. Ragnat A, Parsons, J., Radhakrishnan, R. The barriers determining the use of the carer support subsidy in the Northland region of New Zealand-A qualitative study. *Journal of Geriatric Care and Research* 2018, 5.

2. Jorgensen D, Parsons M, Jacobs S, Arksey H. The New Zealand informal caregivers and their unmet need. *New Zealand Medical Journal* 2010, 123:9-16.

3. Cheung G, Rivera-Rodriguez C, Martinez-Ruiz A, Ma'u E, Ryan B, Burholt V, Bissielo A, Meehan B. Impact of COVID-19 on the health and psychosocial status of vulnerable older adults: study protocol for an observational study. *BMC Public Health* 2020, 20:1814.

4. Sheridan NF, Kenealy TW, Connolly MJ, Mahony F, Barber PA, Boyd MA, Carswell P, Clinton J, Devlin G, Doughty R, et al. Health equity in the New Zealand health care system: a national survey. *International Journal for Equity in Health* 2011, 10:45.

5. Whitehead J, Davie G, de Graaf B, Crengle S, Fearnley D, Smith M, Lawrenson R, Nixon G. Defining rural in Aotearoa New Zealand: A novel geographic classification for health purposes. *New Zealand Medical Journal* 2022, 135:24-40.

6. Fearnley D, Lawrenson R, Nixon G: 'Poorly defined': Unknown unknowns in New Zealand Rural Health. *The New Zealand Medical Journal* 2016, 129:77-81.

7. Census 2023 Hub. 2023 Census population change by age group and RC. Interactive map. [<https://statsnz.maps.arcgis.com/apps/mapviewer/index.html?layers=b20dc2acba8247f0b2924befe4082283&layerId=0>]

8. 2023 Census population counts (by ethnic group, age, and Māori descent) and dwelling counts [<https://www.stats.govt.nz/information-releases/2023-census-population-counts-by-ethnic-group-age-and-maori-descent-and-dwelling-counts/>]
