# Supplementary material for: Understanding the contribution of primary and community services to health system resilience during the COVID19 Pandemic in Aotearoa, New Zealand: a qualitative interview study
Source: BMC Health Serv Res. 2024 Dec 24;24:1650. doi: 10.1186/s12913-024-12078-6 (PMC11667889; doi:10.1186/s12913-024-12078-6)
Supplement: Supplementary file 2 — Additional file 2. [file 12913_2024_12078_MOESM2_ESM.docx]

**ADDITIONAL FILE 2**

**Health Equity & Wellbeing among older people’s caregivers during COVID 19 restrictions**

**Semi-structured Interview Guide for Service Providers**

**SECTION 1**

| **PREAMBLE: We are interested in experiences of caregivers to older people, at the different alert levels during the COVID19 pandemic and whether this was same or different before the pandemic. We would like to discuss your perceptions of caregiving by family and friends who were providing support to an older person, and the resources that your organisation were able to deploy to support caregivers and older care-recipients. I will refer to alert levels 1-4 as ‘COVID19 restrictions’ but I would like you to tell me about any changes across the different levels of restriction when this is appropriate.** | | | | | | | | |
| --- | --- | --- | --- | --- | --- | --- | --- | --- |
| I’d like to start by asking some questions about your role and the organisation that you work for: | | | | | | | | |
|  |  |  |  |  |  |  |  |  |
| 1. What is your job title? _____________ |  |  |  |  |  |  |  |  |
|  |  |  |  |  |  |  |  |  |
| 1. How long (years) have you been in your present role in this organisation? _____________ | | | | |  |  |  |  |
|  |  |  |  |  |  |  |  |  |
| 1. What type of organisation do you work for? |  |  |  | DHB |  | Māori Health |  | Pasifika Health |
|  |  |  |  |  |  |  |  |  |
| Home care provider and/or community support services: | | | | |  | Private (for profit) | | |
|  |  |  |  |  |  |  |  |  |
|  |  |  |  |  |  | Social enterprise | | |
|  |  |  |  |  |  |  |  |  |
|  |  |  |  |  |  | Not for profit/registered charity | | |
|  |  |  |  |  |  |  |  |  |

**SECTION 2**

| **PREAMBLE: You are taking part in this study because your organisation provided information, health care, home care or community support to older people, or caregivers that were providing support to older people during COVID19 restrictions.** | |
| --- | --- |
| **Question** | **Prompts** |
| 1. What is the primary purpose or mission of your organisation? | **Check website prior to interview to see if mission in described.** |
| 1. Before COVID19 restrictions, what services did you provide for older people or caregivers? |  |
| 1. Please describe any new services, resources or outreach activities that you developed specifically to support caregivers or older people during COVID19 restrictions? | Were any specifically to support Māori whānau-based care networks, or Pacific aiga-based care networks, or for PLWD; challenges of English as a second language. **If there were specific hard copy or web-based resources (please ask for a copy to be mailed, emailed or obtain URL)** |
| 1. IF YES, please describe the impact that **each** of these had on caregivers. |  |
| 1. Can you describe any challenges associated with operating or delivering services resources or outreach activities in the way that you wanted to during COVID19 restrictions? | Availability of resources (e.g. PPE) |
| 1. Please describe any operating procedures and strategies that you developed in response to COVID19 restrictions? | To protect staff, to protect clients, shielding advice for workers who had vulnerable family members. |
| 1. How did you maintain relationships with your clients, and communicate with unpaid caregivers? | Telehealth, digital methods of contact, Zoom, social media (FaceBook, Twitter), telephone, radio, TV, leaflets, letters Perceptions of success/challenges. |

**SECTION 3**

| **PREAMBLE: I’d like to discuss your perceptions about unpaid caregiving by family and friends during COVID19 restrictions. I am interested in unpaid caregiving when the care-recipient is an older person. The care and support could be provided either by one person, or by a group of people, such as a family or whānau.** | |
| --- | --- |
| **Question** | **Prompts** |
| 1. Please describe the types of requests for support you received from unpaid caregivers in relation to information, goods/equipment, services, or other unmet needs? |  |
| 1. Please describe your perception or impression of any changes to unpaid caregiving during different levels of COVID19 restrictions that you were made aware of? | Changes in amount (hours per week or day) OR types of emotional/functional support changed OR living arrangements changed and why. How were changes identified (e.g. calls to organisation, seen by staff). Positive or negative impacts on caregivers. |
| 1. Describe any additional out-of-pocket expenditure for unpaid caregivers (compared to before this period) that you were made aware of, and what this was for? | Services, goods (e.g. bedding, mobility aids and incontinence products), home adaptations to improve accessibility, increased heating, laundry, transportation. |
| 1. Could you describe how easy or difficult you think it may have been for unpaid caregivers to navigate health and social care systems during COVID 19 restrictions? | Facilitators and barriers. Apps, online resources and social media, bureaucratic hurdles. Culturally or linguistically safe, appropriate and accessible. Generic resources suitable for people living with dementia. |

**SECTION 4**

| **PREAMBLE: Now I would like to ask you about your perceptions of any influences of community and neighbourhood on unpaid caregiving during COVID19 restrictions** | |
| --- | --- |
| PURPOSE: *The* ***community/ neighbourhood*** *area of enquiry will identify inequities in access to services for caregivers in disadvantaged communities and for those living at a distance from ‘resource centres’. We also need to know more about exclusion from access to information due to a digital divide in access to broadband internet services.* | |
| **Question** | **Prompts** |
| 1. Please describe any organisational challenges that you had to deal with during COVID restrictions that related to providing your services, resources or outreach activities in particular geographic locations, for example, to rural and remote communities, or particularly disadvantaged areas? |  |
| 1. Please describe any particular challenges that you think unpaid caregivers faced that were associated with their geographic location? | Facilities, amenities (e.g. access to supermarket/groceries, takeaways, food delivery services, local medical services, community services), transport. Digital exclusion, mobile coverage, costs, satellite or fibre wifi, quality of connection. |

**SECTION 5**

| **PREAMBLE: Now I would like to ask you about co-ordination of services, and Government information that was issued during COVID19 restrictions, how you accessed this information and whether it met your organisational needs** | |
| --- | --- |
| PURPOSE: *The* ***social structural and cultural*** *area of enquiry will explore whether information and resources developed during the pandemic addressed caregivers’ needs, or whether there are unresolved issues that still need to be tackled in order for us to be prepared for future scenarios. For example, was guidance culturally safe and sensitive for Māori and Pacific caregivers? Were there suitable support systems for caregivers who lived at a distance from care recipients? And were generic resources suitable for caregivers supporting people living with dementia?* | |
| **Question** | **Prompts** |
| 1. Please describe the relationships between your organisation and others that you were in contact with during alert levels 4 and 3, for example, how did you communicate or collaborate during the COVID19 restrictions? | Local and national organisations, DHBs, other providers. |
| 1. Please describe any informational, material (resources) or financial support you received from the Government and Ministries to help you to serve your clients during COVID19 restriction? | Government information, resources. Emails, websites, flyers, TV announcements/media statements. Culturally or linguistically safe, appropriate and accessible. Generic resources suitable for people living with dementia. Level of confidence in the information provided. |
| 1. Was there any support provided by the Government and Ministries during COVID19 restrictions that worked really well for you organisation or clients (i.e. unpaid caregivers/older people), or was especially helpful? |  |
| 1. Was there any other information you think should have been provided during COVID19 restrictions for your organisation or clients (i.e. unpaid caregivers/older people), but wasn’t? |  |
| 1. Before I conclude this interview is there anything else you would like to say about the provision of organisational care and support, or unpaid caregiving during the COVID19 restrictions? |  |
